# NURSING HOME STAFF RISK PERCEPTIONS DURING THE COVID-19 PANDEMIC

**DOI:** 10.1093/geroni/igac059.921

**Published:** 2022-12-20

**Authors:** Joan Carpenter, Nia Kooiman, Kalei Kowalchik, Jacqueline Mogle, Jasmine Newman, Liza Behrens

**Affiliations:** University of Maryland School of Nursing, Baltimore, Maryland, United States; University of Maryland, Baltimore, Maryland, United States; Pennsylvania State University, University Park, Pennsylvania, United States; The Pennsylvania State University, University Park, Pennsylvania, United States; University of Maryland, Baltimore, Maryland, United States; Pennsylvania State University, University Park, Pennsylvania, United States

## Abstract

Nursing home (NH) staffs’ risk perceptions related to NH residents’ health and safety are a barrier to honoring residents’ preferences for care and activities; this was exacerbated during the COVID-19 pandemic. This study intended to describe NH staffs’ self-perceived risk perceptions during the COVID-19 crisis as measured by the validated Risk Propensity Scale. Participants (N=27) included licensed and unlicensed nursing staff (e.g., RN, LPN, CNA), social workers, and activities directors, were mostly female (85%), White (74%), non-Hispanic (93%), and had more than three years of experience working in NHs (78%). Survey results indicated that males considered themselves risk seekers (M=7.5 out of 9) and females risk avoiders (M=4.0). Despite identifying as a risk seeker or risk avoider, the majority of participants agreed with “safety first”, preferred to avoid risk, and viewed risks as “a challenge.” Findings will be discussed considering implications for person-centered risk management during a public health crisis.

